# Genome-Wide Association Study With Growth-Related Traits and Secondary Metabolite Contents in Red- and White-Heart Chinese Fir

**DOI:** 10.3389/fpls.2022.922007

**Published:** 2022-06-30

**Authors:** Sen Cao, Hongjing Duan, Yuhan Sun, Ruiyang Hu, Bo Wu, Jun Lin, Wenjian Deng, Yun Li, Huiquan Zheng

**Affiliations:** ^1^National Engineering Laboratory for Tree Breeding, Key Laboratory of Genetics and Breeding in Forest Trees and Ornamental Plants of Ministry of Education, The Tree and Ornamental Plant Breeding and Biotechnology Laboratory of National Forestry and Grassland Administration, College of Biological Sciences and Technology, Beijing Forestry University, Beijing, China; ^2^Experimental School Affiliated to Chinese Academy of Sciences, Beijing, China; ^3^Experimental Center of Forestry in North China, Chinese Academy of Forestry, Beijing, China; ^4^Longshan State Forest Farm of Lechang, Lechang, China; ^5^Guangdong Provincial Key Laboratory of Silviculture, Protection and Utilization, Guangdong Academy of Forestry, Guangzhou, China

**Keywords:** Chinese fir, secondary metabolite, growth-related traits, single nucleotide polymorphism marker, flavonoid

## Abstract

Chinese fir [*Cunninghamia lanceolata* (Lamb.) Hook] is an important evergreen coniferous tree species that is widely distributed in many southern provinces of China and has important economic value. The Chinese fir accounts for 1/4 and 1/3 of the total artificial forest area and stock volume, respectively. Red-heart Chinese fir is popular in the market because of its high density and red heartwood. The long-growth cycle hindered the breeding process of Chinese fir, while molecular marker-assisted breeding could accelerate it. However, Chinese fir, a perennial conifer species, has a large genome, which has not yet been published. In this study, the growth-related traits and secondary metabolite contents of red- and white-heart Chinese fir were measured and found to be different between them. There are extremely significant differences among growth-related traits (*p* < 0.001), but secondary metabolite contents have different correlations due to differences in chemical structure. Moreover, genotype effect analysis of the substantially correlated single nucleotide polymorphisms (SNPs) revealed that most of the loci related to each growth-related traits were different from each other, indicating a type specificity of the genes regulated different growth-related traits. Furthermore, among the loci related to secondary metabolite contents, nine loci associated with multiple metabolite phenotypes such as Marker21022_4, Marker21022_172, Marker24559_31, Marker27425_37, Marker20748_85, Marker18841_115, Marker18841_198, Marker65846_146, and Marker21486_163, suggesting the presence of pleiotropic genes. This study identified the potential SNP markers associated with secondary metabolites in Chinese fir, thus setting the basis for molecular marker-assisted selection.

## Introduction

Chinese fir is a fast-growing, economical timber species widely distributed in southern China. It is well-known for its high quality and large quantity. In 2021, the artificial forest area and stock volume of Chinese fir both ranked first in China. Since 1976, breeders have been striving to breed Chinese fir varieties of high quality and quantity (Zheng et al., 2013). Among them, red-heart (RH) Chinese fir is very popular because of its red heartwood color, high density, and has higher content of cedarwood and sclareol when compared with the sapwood (Duan et al., 2015; Yang et al., 2016). In our earlier study, it was found that flavonoid metabolites were the main differential metabolites between red- and white-heart Chinese fir heartwood, especially luteolin-7-O-glucuronide-5-O-rhamnoside and luteolin-7-O-glucoside (Cao et al., 2022). Besides, in the previous study, we collected 700 Chinese fir clones, which preserved in the Longshan state forest farm of Guangdong Province. We assessed their genetic diversity, and moreover, constructed a core group of 300 representative clones by using 21 simple sequence repeat markers and wood-property traits with the aim of maximizing representation of the 700 population (Duan et al., 2017).

Metabolites refer to substances produced or consumed by metabolic process, generally including only small molecules. Metabolites are well known for a variety of functions, including defense, structure, fuel, signaling, and interactions with other organisms and themselves (as a cofactor to an enzyme; Fang et al., 2019). Metabolites are divided into primary and secondary metabolites. Unlike primary metabolites, directly involved in normal growth, development, and reproduction, the secondary metabolites often play important roles in plant coloration, resistance, and defense against microbial and insect (Celedon and Bohlmann, 2018). The core primary metabolic pathways are similar in most plant and non-plant species; however, much more secondary metabolites is specifically produced in plants (Alseekh and Fernie, 2018). The total number of plant metabolites is expected to exceed 200,000 right now, most of which are secondary metabolites or their derivatives (Schwab, 2003; Pichersky and Lewinsohn, 2011). Secondary metabolites in plants play a critical role in their interactions with the environment.

**Figure 1:**
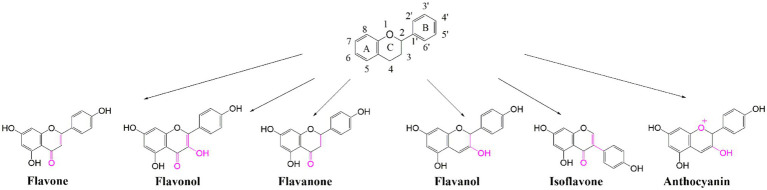
Chemical structures and classification of flavonoids. The skeleton structure consists of A, B, and C rings with groups positions numbered. Flavonoids are sub-divided according to the hydroxylation pattern and conjugation between the aromatic rings (indicated in purple), and the chemical structures of six simple flavonoids are showed.

Among the secondary metabolites, polyphenolic metabolites are the most abundant and widely distributed in nature (Tohge et al., 2013). Flavonoids, as the most abundant kind of polyphenols, were estimated to contain more than 8,000 compounds, which play important roles in many biological processes, such as resistance to biotic/abiotic stress (Winkelshirley, 2002) and some chronic diseases, including cardiovascular diseases and certain cancers (Luo et al., 2008). Flavonoids are composed of a common C6-C3-C6 structure with two benzene rings (ring A and ring B) interconnected by a three-carbon heterocyclic pyran ring (ring C), which can be subdivided into six categories according to their chemical structures, namely flavones, flavonols, flavanones, flavanols, isoflavones, and anthocyanins (Figure 1), with a total of more than 5,000 species (Peng et al., 2017). Flavonoids and their derivatives are primarily synthesized by a variety of enzymes involved in phenylalanine and flavonoid biosynthesis pathways. Phenylalanine synthesized in the shikimate pathway is cleaved by phenylalanine ammonia lyase to generate trans-cinnamic acid, which can be used to synthesize lignin, lignans, and flavonoids (Fraser and Chapple, 2011; Maeda and Dudareva, 2012). Subsequently, a cytochrome 450 monooxygenase, catalyzed by cinnamic acid 4-hydroxylase hydroxylates the C4 position of cinnamic acid to produce 4-coumaric acid. Naringenin chalcone is synthesized by 4-coumarate CoA ligase and catalyzed by ATP consumption. It is used as a substrate to synthesize flavonoids and their derivatives by chalcone synthase, chalcone isomerase, flavonoid 3-hydroxylase, flavonoid synthase, flavonoid 3′-hydroxylase, flavonoid 3′5’-hydroxylase, flavonoid synthase I, and UDP-glycosyltransferase.

Plants in nature contain only certain classes of metabolites, so most plant metabolites show species-specific accumulation (Fernie et al., 2004). The diversity of metabolites within plants makes them ideal models for dissecting the genetic basis of metabolite accumulation. Metabolic pathways have been dissected in several ways, including screening mutant libraries (Yonekura-Sakakibara et al., 2014), analyzing gene families (Sadre et al., 2016), and conducting comparative genomics studies (Huang et al., 2017). To gain a deeper understanding of the genetic and biochemical basis of diversity in the metabolome and to artificially synthesis natural metabolites, next-generation sequencing-based linkage and association maps have been applied to metabolomic studies (Luo, 2015; Alseekh and Fernie, 2018). Not only that the genome-wide association studies (GWASs) has also been applied to the study of plant metabolome diversity due to the development of genome sequencing technologies (Fang and Luo, 2019). Metabolite-based GWAS have the advantage of greatly improving large-scale gene-metabolite interactions, annotation and identification in plants, and provide insights into the basic genetic studies of plant metabolome (Chen et al., 2014). Metabolite-based GWAS has now become one of the most using tools for identifying genetic determinants of plant metabolic diversity (Fang and Luo, 2019).

Metabolites are considered to be the bridge between the genome and phenotype and, in some cases, can be the cause or marker of morphological features (Saito, 2013). As a result, increasing interest has been focused on studying the association of metabolites, an intermediate trait associated with the biochemical and physiological states of plants (Chen et al., 2014; Wen et al., 2014). Plant height and grain shape, as complex traits, are generally determined by many loci with less impact (Huang et al., 2010), while metabolite content, especially secondary metabolite content, is usually regulated by a few more influential loci (Chen et al., 2014). Plant primary and secondary metabolites have been extensively studied, and hundreds of quantitative trait loci have been detected in *Arabidopsis*, rice, and tomato (Keurentjes et al., 2006; Schauer et al., 2006; Chan et al., 2010; Matsuda et al., 2012). Association analysis of core oil biosynthesis with single nucleotide polymorphism (SNP) loci found that 74 of 1 million loci in 368 core clones were significantly associated with grain oil concentration and fatty acid composition (*p* < 1.8E-6; Li et al., 2013). Molecular marker assisted breeding selection is based on that the strong association of the molecular markers with the target trait genes. By identifying molecular markers, the genes responsible for the target trait can also be detected.

In this study, we used an existing core population of 300 Chinese fir clones, including 48 RH and 252 white-heart (WH) Chinese fir, to examine both phenotypes, such as growth-related traits and secondary metabolite content in heartwood, and genetic polymorphisms to identify correlations between RH and WH Chinese fir. The identification of genetic polymorphisms and phenotypes correlation loci provides a theoretical basis for future research on RH Chinese fir metabolites and lays a foundation for molecular marker assisted breeding of RH Chinese fir.

## Materials and Methods

### Plant Materials

Three hundred Chinese fir clones were collected from the *ex-situ* gene bank of the Longshan State Forest Farm, Guangdong Province, China (25°11′N, 113°28′E, 285–296 m above sea level). Six growth-related traits and six secondary metabolite contents in the heartwood were measured in all 300 clones, with at least three randomly selected ramets per clone. Growth-related traits included tree height (H), diameter at breast height (DBH), stem volume (V), percentage of heartwood (P), wood basic density (WBD), and hygroscopicity (HY). The secondary metabolites included dihydrokaempferol (DK), dihydroquercetin (DQ), luteolin, pinocembrin, apigenin, and naringenin. Three individuals were selected as biological replicates.

### Growth-Related Traits and Secondary Metabolites Detection

H and DBH were measured during the field surveys and V, P, WBD, and HY were measured in the laboratory using methods described by Duan et al. (2016). *V* = 0.000058777042 × *DBH*^1.9699831^ × *H*^0.89646157^. *P* was measure by using the formula *P* = *r*^2^/*R*^2^ × 100%, which *r* and *R* represent the length of heartwood and xylem, respectively. WBD and HY were measured using formula WBD = 1/(*W*1/*W*2 − 0.346) and HY = (*W*1 − *W*2)/*W*2, which *W*1 and *W*2 represent the water-saturated weight and the oven-dry weight, respectively. The contents of six secondary metabolites, including DK, DQ, luteolin, pinocembrin, apigenin, and naringenin were measured by using liquid chromatography with mass spectrometry (LC-MS/MS), and the detailed methods and parameters of LC-MS/MS were described by Cao et al. (2022). *V_G_* (genetic variance), *V_E_* (environment variance), and residual were measured by using SPSS 24.0, and *H_B_*^2^ (generalized heritability) was calculated by using formula below:


$$H_{B}^{2}=\frac{V_{G}}{V_{G}+V_{E}+\mathrm{residual}}\times 100\%$$

### DNA Extraction and Library Construction

Three-hundred microgram of Chinese fir needles were used for DNA extraction. The DNA quality and concentration of the individuals in the group were detected, the DNA quality was detected by 1% agarose gel, and the electrophoresis results were observed and recorded by the gel imaging system, and stored in an ultra-low temperature refrigerator.

At present, no genome sequence information of Chinese fir has been released, and there are no genomes of other closely related species for reference, while the conifer species that have released genome sequences are white spruce, Norway spruce, loblolly pine and Chinese pine (Birol et al., 2013; Nystedt et al., 2013; Neale et al., 2014; Niu et al., 2022), therefore, based on the genome size and GC content of Chinese fir, the Norway spruce genome was finally selected as the reference genome for electronic digestion prediction. The Norway spruce (*Picea abies*) genome was used as a reference for electronic digestion. The assembled genome size was 12Gb, and the GC content was 37.87%. The download address is: http://congenie.org/.

### SNPs Marker Development and Locus-Phenotype Association Study

Using the deepest sequence in each SLAF tag as the reference sequence, the reads obtained by sequencing were aligned to the reference sequence, and SNPs were developed using two software, GATK (McKenna et al., 2010) and SAMtools (Li et al., 2009). The intersection of SNP markers obtained by the two methods was used as the final reliable SNP marker dataset, according to the integrity ≥0.5, the minor genotype frequency (MAF) ≥ 0.05, the obtained SNPs were filtered, and finally the population SNPs with high quality and good consistency were obtained for further analysis.

Based on high-quality SNPs, association analysis was performed on growth-related traits and flavonoid contents traits of associated populations using the mixed linear model (MLM) of TASSEL 5.0 software (Bradbury et al., 2007). The formula is calculated as follows:


Y=u+Xα+Qβ+Kμ+e

MLM uses *Q* + *K*, which is a comprehensive analysis method of population structure and kinship, and substitutes the kinship coefficient matrix into the model as a random effect. The influence of other factors on the association results. The genotype vector is used as *X*, the phenotype value vector is used as *Y*, the fixed effect value vector is used as *u*, *α* is the weight vector for each marker, *β* is the trait vector for each subgroup, *μ* is the association matrix, and *e* is the residual. MLM correction will be more stringent, so it also has a relatively high accuracy, reducing the occurrence of false positives, but some useful SNP sites may be lost due to too high standards.

## Results

### Variation in Growth-Related Traits and Secondary Metabolite Content in Heartwood

In this study 300 Chinese fir clones, including 48 RH and 252 WH Chinese fir, were analyzed. First, H, DBH, V, P, WBD, and HY were measured (Table 1). In RH, the H, DBH, and HY were 7.24 m, 12.64 cm, and 237.65%, respectively, which resulted lower than those obtained from the WH (8.08 m, 13.72 m, and 263.92%). In contrast, the V, P, and WBD in RH were 0.0620 m^3^, 22.26%, and 0.3393 g.cm^−3^, respectively, which resulted higher than those measured from the WH. Moreover, the amplitude of all six growth-related traits in RH were 4.50–11.67 (H), 5.77–21.73 (DBH), 0.0093–0.2273 (V), 13.43–40.61 (P), 0.2512–0.4783 (WBD), and 152.98–333.62 (HY), with the ratios of the maximum to the minimum being lower than those calculated for the WH, thus demonstrating that RH Chinese fir grows more evenly.

**Table 1:** Differences in growth-related traits of red- and white-heart Chinese fir.

		H/m	DBH/cm	V/m^3^	P/%	WBD/g.cm^−3^	HY/%
Average	RH	7.24 ± 0.25	12.64 ± 0.45	0.0620 ± 0.0062	22.26 ± 0.91	0.3393 ± 0.0069	237.65 ± 5.81
	WH	8.08 ± 0.13	13.72 ± 0.21	0.0807 ± 0.0034	21.53 ± 0.40	0.3110 ± 0.0025	263.92 ± 2.55
Amplitude	RH	4.50 ~ 11.67	5.77 ~ 21.73	0.0093 ~ 0.2273	13.43 ~ 40.61	0.2512 ~ 0.4783	152.98 ~ 333.62
	WH	3.25 ~ 14.67	5.25 ~ 24.27	0.0069 ~ 0.3235	8.33 ~ 45.56	0.2286 ~ 0.5102	130.60 ~ 372.27

H, tree height; DBH, diameter at breast height; V, stem volume; P, percentage of heartwood; WBD, wood basic density; HY, hygroscopicity; RH, red-heart Chinese fir, and WH, white-heart Chinese fir.

Furthermore, the content of the following metabolites was examined in both the RH and WH: DK, DQ, luteolin, pinocembrin, apigenin, and naringenin (Table 2). DK, DQ and naringenin in RH were 0.1831, 0.1425, and 3.7770 μg.g^−1^, respectively, which resulted higher than contents of the three metabolites in WH (0.1021, 0.1385, and 2.5778 μg.g^−1^). However, the contents of luteolin, pinocembrin, and apigenin in RH were 0.0485, 0.7882, and 0.0968 μg.g^−1^, respectively, which were lower than those in WH (0.0523, 1.6461, and 0.2124 μg.g^−1^). Among them, the contents of pinocembrin and apigenin in WH were more than 2-fold higher when compared with their content in RH. The amplitude of all six secondary metabolites showed a similar pattern to growth-related traits, indicating that the variation range of RH was lower than that of WH.

**Table 2:** Differences in the content of secondary metabolites in the heartwood of red- and white-heart Chinese fir.

		DK/ug.g^−1^	DQ/ug.g^−1^	Luteolin/ug.g^−1^	Pinocembrin/ug.g^−1^	Apigenin/ug.g^−1^	Naringenin/ug.g^−1^
Average	RH	0.1831 ± 0.0372	0.1425 ± 0.0315	0.0485 ± 0.0087	0.7882 ± 0.1762	0.0968 ± 0.0176	3.7770 ± 0.6335
	WH	0.1021 ± 0.0140	0.1385 ± 0.0274	0.0523 ± 0.0053	1.6461 ± 0.3418	0.2124 ± 0.0438	2.5778 ± 0.2335
Amplitude	RH	0 ~ 1.1838	0 ~ 1.2153	0 ~ 0.3021	0.0128 ~ 6.7513	0 ~ 0.6175	0.1151 ~ 20.1582
	WH	0 ~ 1.7615	0 ~ 5.1900	0 ~ 0.8213	0 ~ 41.8735	0 ~ 0.5283	0.0047 ~ 25.5447

DK, dihydrokaempferol; DQ, dihydroquercetin; RH, red-heart Chinese fir; and WH, white-heart Chinese fir.

The variance components of each trait in the associated population are analyzed and showed in Supplementary Table S1. It was found that *V_E_* of each trait was much lower than *V_G_*. The generalized heritability values were greater than 40% except P, with only 15.15%.

### Correlations Between Pairs of Traits

**Figure 2:**
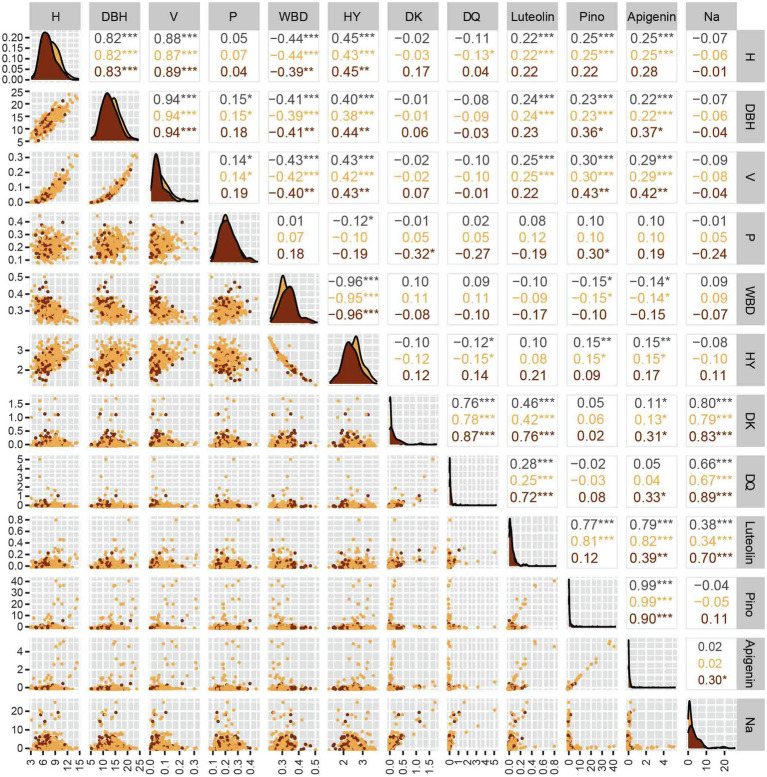
Phenotypic differences and correlations of six growth-related traits and the contents of six secondary metabolites in red-heart and white-heart Chinese fir. H, tree height; DBH, diameter at breast height; V, volume; P, percentage of heartwood; WBD, wood basic density; HY, water absorption; DK, dihydrokaempferol; DQ, dihydroquercetin; Pino: pinocembrin; and Na, Naringenin.

The overall correlation analysis of the selected 12 associated phenotypes is shown in Figure 2. A significant positive correlation among H, DBH, V, and HY was detected, whereas H, DBH, V, and HY were significantly negatively correlated with WBD (*p* < 0.001). Furthermore, no significant difference between WH and RH was observed. We detected high correlation coefficients (*r* > 0.80) among H, DBH, and V. Regardless of growth-related traits or secondary metabolite contents, there was no significant correlation between P and the other 11 traits, and the correlation coefficient (*r*) was only approximately 0.1. A significant negative correlation between WBD and the other growth-related traits, except P, was detected, indicating that the faster the growth of Chinese fir, the lower the density of wood, and the correlation coefficient with HY was the largest (*r* > 0.96). In the correlation analysis of H, DBH, and V, luteolin, pinocembrin, and apigenin showed the same trends. Although the contents of the three flavonoid metabolites were positively correlated with the three growth traits (*p* < 0.001), the correlation coefficient was low (*r* ≈ 0.25), indicating the lack of a linear correlation.

In addition, there were some correlation differences in the secondary metabolite contents (Figure 2). In general, the correlation of secondary metabolites was divided into two categories: an extremely significant positive correlation among DK, DQ, and naringenin, and an extremely significant positive correlation among luteolin, pinocembrin, and apigenin. Specifically, the contents of luteolin and the other five metabolites were significantly different, among which, there was a linear correlation between luteolin and DK (*r* = 0.76), DQ (*r* = 0.72), and naringenin (*r* = 0.70) only in RH. The higher correlation coefficients observed in RH as compared with those in WH suggest that a linear correlation between the compounds exists only in RH. Moreover, DQ was only significantly positively correlated with DK, luteolin, and naringenin, and the correlation coefficient in RH was significantly higher than that in WH, which also showed a linear correlation between these compounds in RH. In addition, there were extremely significant positive correlations between apigenin and pinocembrin only with luteolin and no significant correlations with other metabolites. There were different degrees of correlation between naringenin and the other five metabolites, but the correlation coefficient between naringenin and other metabolites in RH was significantly higher than that in WH, indicating that naringenin is more linearly related to other secondary metabolites in RH.

### GWAS of Traits of Chinese Fir

In our earlier study, simplified genome sequencing of 300 Chinese fir core populations was performed using the SLAF-seq method, and 955,503 SNPs were detected. The obtained population SNPs were further filtered according to integrity ≥0.5 and MAF ≥ 0.05, and finally 166,646 high-quality SNP markers were developed (Duan et al., 2017). The SNP information statistics of each sample were shown in Supplementary Table S2. Six growth-related traits and six flavonoid secondary metabolite contents were measured, and then TASSEL5.0 software mixed linear models (MLM) were used to detect associations between SNP marker loci and traits.

**Figure 3:**
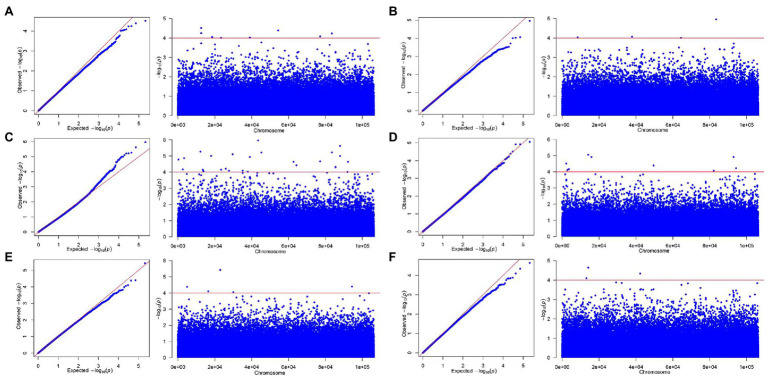
The qq-plot and manhattan of genome-wide association studies for growth-related traits based on mixed linear model (MLM). **(A)** H, **(B)** DBH, **(C)** V, **(D)** P, **(E)** WBD, and **(F)** HY.

**Figure 4:**
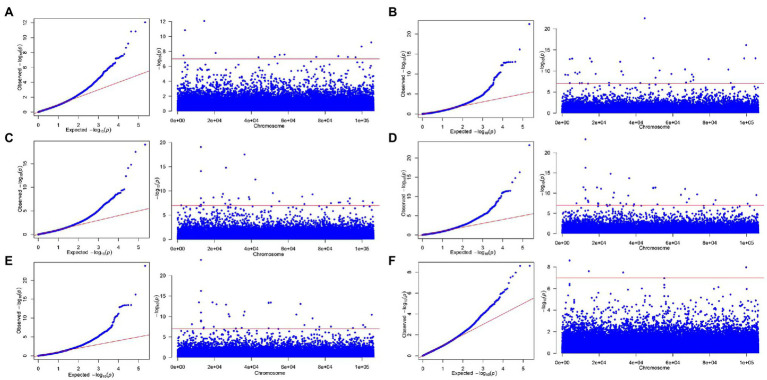
The qq-plot and manhattan of genome-wide association studies for secondary metabolite contents based on MLM. **(A)** Luteolin, **(B)** pinocembrin, **(C)** apigenin, **(D)** naringenin, **(E)** DK, and **(F)** DQ.

Through the qq plot, it was found that the x-axis was a uniformly distributed value (theoretical value) and had undergone −log10 transformation, and the *y*-axis was the actual value of *p* (observed value) and had undergone −log10 transformation. It was caused by random drift, and then begins to deviate from the straight line due to the existence of random drift and the real site, indicating that the association analysis based on the MLM model was suitable for this study (Figures 3, 4). The Manhattan plot showed the inability of the simplified genomic analysis to assign SNP loci to a certain chromosome. Furthermore, in the Manhattan plot, the higher the point, the more significant the association between the marker loci and traits.

Under the significance criterion of *p* < 1E-4, 62 SNP marker loci were detected that were significantly associated with each growth-related trait using a MLM. Under MLM, 9, 4, 32, 5, 9, and 3 SNP markers were significantly associated with H, DBH, V, P, WBD, and HY, respectively (Table 3). On average, there were 10 SNP markers that were significantly associated with each growth-related traits, with the most were associated with V. Statistical analysis of the association interpretation rate of growth-related traits revealed that the overall rate varied from 6.480% (Marker19980_201_SNP associated with DBH) to 12.141% (Marker49894_207_SNP associated with V), with an average interpretation rate of 8.023%. Among them, the SNP markers associated with H accounted for 7.207%–8.659% of phenotypic variation, the SNP markers associated with DBH accounted for 6.480%–9.244% of phenotypic variation, and the SNP markers associated with V accounted for 6.491%–12.141%, the SNP markers associated with P was 6.750%–10.239%, the SNP markers associated with WBD was 6.724%–9.169%, and the SNP markers associated with HY ranged from 6.899% to 7.714%.

**Table 3:** SNPs with significant association to growth-related traits based on MLM genome-wide association study (GWAS).

Trait	Locus	SNP position	Major allele	Minor allele	Minor allele freq.	*p*	*R*^2^ (%)	Add. effect	Dom. effect
H	Marker21013	78	G	A	0.203	3.082E-05	7.725	—	−1.488
	Marker33168	172	C	A	0.083	4.114E-05	7.719	1.545	−2.103
	Marker21013	203	C	T	0.190	5.660E-05	7.207	—	−1.541
	Marker46641	94	C	T	0.062	5.859E-05	8.124	—	−2.562
	Marker42910	4	G	A	0.140	8.232E-05	7.871	—	1.906
	Marker22578	168	A	G	0.177	8.769E-05	8.659	—	—
	Marker22578	120	A	G	0.177	9.301E-05	8.562	—	—
	Marker28223	132	C	T	0.055	9.452E-05	8.560	−1.626	—
	Marker23810	90	G	A	0.075	9.821E-05	8.277	—	—
DBH	Marker46641	94	C	T	0.062	1.104E-05	9.244	—	−4.413
	Marker27912	9	G	A	0.135	8.600E-05	6.711	—	−2.712
	Marker19980	201	C	T	0.323	9.324E-05	6.480	—	—
	Marker36988	54	C	T	0.353	9.830E-05	8.946	—	2.230
V	Marker29658	96	T	C	0.053	1.076E-06	9.692	0.096	—
	Marker49894	207	C	T	0.087	2.397E-06	12.141	—	0.063
	Marker20915	175	T	G	0.115	5.402E-06	8.502	0.124	−0.134
	Marker46641	94	C	T	0.062	6.031E-06	9.628	−0.038	−0.067
	Marker30182	29	G	A	0.162	6.145E-06	8.425	0.047	−0.050
	Marker25513	81	G	T	0.088	7.836E-06	8.209	−0.066	—
	Marker22437	36	C	T	0.045	1.005E-05	9.643	−0.059	—
	Marker49894	179	C	T	0.090	1.007E-05	10.707	—	0.057
	Marker28223	132	C	T	0.055	1.161E-05	9.991	−0.046	—
	Marker18675	17	C	T	0.100	1.377E-05	8.329	−0.050	—
	Marker17912	93	G	A	0.088	1.655E-05	7.892	0.054	—
	Marker17912	199	C	T	0.090	1.672E-05	7.884	−0.054	—
	Marker42910	4	G	A	0.140	2.163E-05	9.131	—	0.053
	Marker36243	4	A	G	0.052	2.298E-05	7.564	−0.057	−0.069
	Marker53909	123	G	A	0.045	2.311E-05	8.274	—	—
	Marker36900	140	C	A	0.083	3.809E-05	7.245	0.048	—
	Marker47941	10	C	T	0.160	4.788E-05	7.669	−0.026	—
	Marker47941	173	G	A	0.162	5.077E-05	7.667	0.026	—
	Marker24491	79	A	G	0.052	6.178E-05	6.717	—	—
	Marker18483	203	C	T	0.060	6.810E-05	6.882	−0.117	—
	Marker21231	160	C	A	0.052	6.985E-05	6.862	0.049	—
	Marker23810	90	G	A	0.075	7.077E-05	8.673	—	—
	Marker27099	140	G	C	0.045	8.231E-05	10.500	—	0.084
	Marker24960	126	G	A	0.053	8.283E-05	6.660	0.068	—
	Marker21313	181	A	G	0.257	8.687E-05	6.507	−0.116	−0.117
	Marker21313	9	A	C	0.262	8.712E-05	6.504	−0.116	−0.117
	Marker21313	17	T	C	0.238	8.734E-05	6.503	0.116	−0.117
	Marker21313	189	T	C	0.253	8.847E-05	6.493	0.116	−0.117
	Marker21313	64	T	C	0.258	8.867E-05	6.492	0.116	−0.116
	Marker21313	173	T	A	0.253	8.875E-05	6.491	0.116	−0.116
	Marker27912	9	G	A	0.135	9.428E-05	6.636	—	−0.042
	Marker53530	32	C	T	0.130	9.736E-05	7.490	−0.028	—
P	Marker23679	33	T	C	0.133	3.760E-06	8.812	0.045	−0.062
	Marker56607	92	G	T	0.040	4.001E-05	10.239	—	—
	Marker19085	164	T	A	0.050	4.150E-05	7.328	0.061	−0.079
	Marker22039	169	G	A	0.190	7.798E-05	8.072	0.027	—
	Marker25629	169	G	A	0.153	8.797E-05	6.750	0.054	—
WBD	Marker21403	124	C	T	0.363	8.812E-06	8.382	—	—
	Marker54715	36	C	T	0.057	1.205E-05	9.169	—	—
	Marker21879	91	A	G	0.067	1.225E-05	9.115	—	0.048
	Marker18373	74	G	A	0.133	3.084E-05	7.386	0.032	−0.041
	Marker31544	181	C	T	0.262	4.068E-05	8.828	—	0.032
	Marker56465	203	T	C	0.390	5.986E-05	7.753	0.015	—
	Marker18749	33	A	C	0.100	6.851E-05	6.916	−0.042	—
	Marker18527	205	T	C	0.067	7.838E-05	6.724	0.094	−0.098
	Marker45838	70	G	A	0.187	8.613E-05	8.185	0.018	—
HY	Marker21403	124	C	T	0.363	2.298E-05	7.714	—	—
	Marker29252	119	A	T	0.478	4.586E-05	7.078	—	−0.218
	Marker21169	24	C	T	0.135	7.999E-05	6.899	—	0.340

*p* < 1E-4.

Under the significant criterion of *p* < 1E-7, using MLM, a total of 163 SNP marker locus were detected that significantly associated with the content of various flavonoid metabolites. Under MLM, 15, 5, 40, 32, 37, and 34 SNP markers were significantly associated with DK, naringenin, DQ, luteolin, pinocembrin, and apigenin, respectively (Table 4), On average, there were 27 significantly associated SNP markers for each growth-related trait, with the most SNP markers associated with DQ. Statistical analysis of the associated interpretation rate of metabolite content revealed that the overall interpretation rate varied from 11.513% (Marker21373_159_SNP associated with taxifolin) to 44.750% (Marker21022_4_SNP associated with apigenin), with an average interpretation rate of 17.833%, higher than the results of association analysis related to growth-related traits. Among these markers associated with Chinese fir growth-related traits, 41 markers were associated with multiple traits, of which 27 were associated with the content of two metabolites and 14 were associated with the content of three metabolites, which was significantly higher than the results of the association analysis of growth-related traits, indicating that the genes regulating flavonoid metabolites were relatively concentrated. Correlation analysis between phenotypic traits also showed that there was a certain degree of correlation between the tested traits, suggesting the presence of pleiotropic genes, and the phenotypic correlation was verified at the molecular level. These SNPs jointly control different phenotypic traits and can be used as key molecular markers in future resource identification, tree selection, and other studies.

**Table 4:** SNPs with significant association to flavonoid contents based on MLM GWAS.

Trait	Locus	SNP position	Major allele	Minor allele	Minor allele freq.	*p*	*R*^2^ (%)	Add. effect	Dom. effect
DK	Marker21486	163	G	A	0.050	8.510E-13	20.719	0.835	−0.873
	Marker18841	115	T	C	0.042	1.464E-11	23.326	—	0.734
	Marker18841	198	A	C	0.042	1.487E-11	23.327	—	0.733
	Marker125630	139	C	T	0.315	6.191E-10	15.297	−0.526	−0.521
	Marker65846	146	C	T	0.040	2.228E-09	18.153	—	1.059
	Marker23099	68	C	T	0.077	1.611E-08	13.515	−0.292	−0.329
	Marker34490	193	T	A	0.040	2.679E-08	14.325	—	0.518
	Marker33496	159	T	A	0.063	2.920E-08	15.636	0.269	—
	Marker18633	199	C	T	0.075	3.494E-08	12.263	−0.321	—
	Marker49264	89	G	C	0.102	4.346E-08	12.823	—	0.311
	Marker32593	75	C	T	0.107	5.324E-08	12.043	−0.363	—
	Marker41923	188	A	G	0.048	5.560E-08	12.332	—	—
	Marker54012	78	G	A	0.067	5.634E-08	13.503	0.207	—
	Marker29741	173	T	C	0.057	6.111E-08	13.283	0.280	—
	Marker59430	131	G	A	0.305	6.165E-08	11.943	0.381	−0.368
Naringenin	Marker18841	115	T	C	0.042	2.461E-09	21.009	—	11.679
	Marker18841	198	A	C	0.042	2.525E-09	21.016	—	11.675
	Marker65846	146	C	T	0.040	1.069E-08	16.023	—	17.050
	Marker21486	163	G	A	0.050	2.433E-08	12.664	11.514	−12.322
	Marker26476	16	G	A	0.062	3.173E-08	12.445	4.387	—
DQ	Marker30018	68	C	T	0.062	3.400E-23	43.549	−2.531	−2.461
	Marker65846	146	C	T	0.040	7.281E-17	39.703	—	2.649
	Marker31516	174	G	A	0.055	9.029E-14	22.394	1.589	−1.653
	Marker114249	12	C	T	0.345	1.008E-13	22.296	−1.592	−1.624
	Marker63437	129	T	C	0.162	1.046E-13	22.226	1.611	−1.584
	Marker21656	117	C	T	0.050	1.057E-13	22.227	−1.600	−1.646
	Marker19203	80	C	T	0.052	1.169E-13	38.373	−1.616	−1.582
	Marker44155	177	C	T	0.047	1.556E-13	22.334	−1.603	−1.623
	Marker18877	38	T	G	0.060	1.572E-13	22.420	1.605	−1.610
	Marker18877	37	G	A	0.060	1.593E-13	22.427	1.604	−1.610
	Marker21913	171	T	C	0.087	6.035E-13	21.035	1.050	−1.083
	Marker21913	177	T	C	0.072	6.424E-13	20.976	1.050	−1.075
	Marker26013	89	C	T	0.042	6.660E-13	24.223	−1.600	−1.656
	Marker37949	153	G	C	0.047	3.810E-11	18.003	−1.421	1.646
	Marker37949	176	T	C	0.050	4.536E-11	17.841	−1.423	1.639
	Marker51728	151	C	T	0.065	4.648E-11	17.321	−0.974	−1.085
	Marker32631	80	G	A	0.050	7.677E-11	17.206	0.551	—
	Marker26201	186	T	A	0.057	1.144E-10	16.789	−1.441	1.604
	Marker26201	154	C	T	0.058	1.330E-10	16.649	1.443	1.598
	Marker20250	167	T	C	0.078	2.087E-10	16.143	0.970	−0.960
	Marker19608	125	C	T	0.057	2.394E-10	16.065	−0.551	—
	Marker19937	182	C	T	0.050	4.826E-10	15.495	−0.937	−0.990
	Marker22489	171	G	A	0.058	5.026E-10	15.473	0.955	−0.944
	Marker34953	138	A	C	0.043	5.254E-10	15.832	−0.935	−1.012
	Marker39702	171	C	T	0.135	7.023E-10	15.254	1.495	1.446
	Marker18265	135	G	A	0.080	7.813E-10	15.727	−1.475	1.528
	Marker26476	16	G	A	0.062	8.310E-10	17.778	0.483	—
	Marker18627	52	A	G	0.060	9.157E-10	15.048	1.479	1.523
	Marker40064	149	A	G	0.053	2.400E-09	14.318	−0.581	−0.708
	Marker31911	173	G	T	0.160	4.002E-09	13.869	−0.446	−0.507
	Marker40064	36	C	T	0.058	1.392E-08	12.960	−0.499	—
	Marker39326	88	G	A	0.077	2.981E-08	12.526	0.416	—
	Marker38187	131	A	G	0.095	5.437E-08	11.911	−0.494	—
	Marker24343	185	C	T	0.057	6.261E-08	11.831	−0.450	—
	Marker53040	195	C	T	0.067	7.037E-08	16.948	−0.746	—
	Marker29286	158	C	G	0.045	7.343E-08	13.304	—	1.397
	Marker20556	64	G	A	0.060	7.643E-08	12.387	—	0.816
	Marker18877	44	G	A	0.110	7.666E-08	11.864	0.779	−0.806
	Marker20263	178	C	T	0.088	8.055E-08	11.719	−0.342	—
	Marker21373	159	G	C	0.048	9.968E-08	11.513	0.791	−0.893
Luteolin	Marker21022	4	G	C	0.123	8.751E-20	36.046	0.204	−0.218	
	Marker27425	37	C	T	0.053	3.023E-18	31.360	−0.390	−0.402	
	Marker24559	31	G	C	0.058	1.620E-15	25.680	0.249	−0.239	
	Marker21022	172	G	A	0.122	8.174E-15	28.545	0.199	−0.211	
	Marker29230	53	A	G	0.067	4.440E-13	21.062	−0.183	−0.203	
	Marker32230	180	G	A	0.070	2.709E-10	16.009	0.193	−0.216	
	Marker24128	170	T	G	0.080	3.970E-10	15.756	0.196	−0.180	
	Marker40944	148	A	G	0.058	4.927E-10	16.273	−0.124	—	
	Marker38381	165	T	A	0.070	1.426E-09	15.013	0.189	−0.200	
	Marker23996	145	C	T	0.067	1.678E-09	14.588	−0.188	−0.176	
	Marker28138	127	C	A	0.167	1.826E-09	14.703	0.187	−0.181	
	Marker22793	161	T	A	0.050	2.163E-09	14.356	0.184	−0.197	
	Marker21022	171	A	G	0.327	3.147E-09	14.743	−0.089	−0.090	
	Marker26477	93	T	C	0.055	3.271E-09	14.035	0.188	−0.183	
	Marker55321	88	C	T	0.063	3.404E-09	17.896	—	0.272	
	Marker28210	173	G	A	0.055	4.789E-09	13.877	0.183	−0.192	
	Marker25068	129	G	T	0.037	7.560E-09	17.499	—	—	
	Marker68088	184	G	A	0.040	1.232E-08	17.923	0.093	—	
	Marker54016	64	C	T	0.042	1.321E-08	15.768	—	—	
	Marker33425	154	C	T	0.103	1.352E-08	13.118	−0.126	—	
	Marker50002	65	C	T	0.062	2.283E-08	13.685	—	0.199	
	Marker21402	41	C	T	0.170	2.367E-08	12.565	−0.093	−0.100	
	Marker49379	44	G	A	0.070	2.385E-08	17.073	—	0.162	
	Marker405384	147	C	T	0.100	2.510E-08	12.875	−0.143	—	
	Marker46448	143	T	G	0.047	3.198E-08	15.351	0.101	—	
	Marker55142	76	G	A	0.037	3.879E-08	15.765	0.090	—	
	Marker24465	5	T	G	0.173	4.147E-08	12.112	0.101	—	
	Marker38907	186	G	A	0.060	5.006E-08	12.674	0.109	—	
	Marker25236	139	C	T	0.083	5.823E-08	11.816	—	−0.156	
	Marker21022	197	A	G	0.388	6.429E-08	12.339	—	−0.080	
	Marker21022	162	A	G	0.385	6.936E-08	12.291	—	−0.079	
	Marker18306	48	C	T	0.065	8.840E-08	11.589	−0.117	—
Pinocembrin	Marker21022	4	G	C	0.123	4.636E-24	43.676	14.520	−14.462	
	Marker21022	172	G	A	0.122	5.250E-17	34.128	13.436	−13.362	
	Marker24559	31	G	C	0.058	1.651E-15	25.673	15.647	−15.068	
	Marker27425	37	C	T	0.053	2.071E-14	23.669	−20.537	−21.080	
	Marker20748	85	G	A	0.057	3.505E-12	19.408	18.548	−17.349	
	Marker22660	151	C	T	0.058	4.017E-12	21.044	−18.597	−17.421	
	Marker31865	188	T	G	0.052	4.349E-12	25.647	18.292	−19.530	
	Marker31467	166	G	A	0.053	4.856E-12	19.117	18.595	−17.769	
	Marker31467	190	G	C	0.088	6.091E-12	18.936	18.525	−18.200	
	Marker23202	152	G	C	0.062	8.444E-12	21.841	18.316	−19.416	
	Marker37949	139	G	A	0.045	9.259E-12	27.197	18.482	−18.231	
	Marker21022	171	A	G	0.327	1.500E-11	18.262	−6.189	−6.305	
	Marker25068	129	G	T	0.037	7.452E-11	21.215	—	11.560	
	Marker40944	148	A	G	0.058	2.007E-10	16.894	−7.884	−8.397	
	Marker36953	169	C	G	0.057	2.566E-10	16.087	−17.857	−18.780	
	Marker166717	48	G	A	0.058	2.843E-10	15.993	17.886	−18.533	
	Marker27288	176	A	G	0.058	3.928E-10	16.225	−17.927	−17.670	
	Marker24949	197	T	G	0.080	1.194E-09	14.796	11.766	−12.438	
	Marker21022	162	A	G	0.385	1.753E-09	14.537	−4.870	−5.382	
	Marker21022	118	C	T	0.388	1.906E-09	14.462	−4.817	−5.387	
	Marker21022	197	A	G	0.388	2.324E-09	14.309	−4.769	−5.314	
	Marker38907	186	G	A	0.060	2.440E-09	14.705	7.368	—	
	Marker46448	143	T	G	0.047	5.163E-09	15.900	6.687	—	
	Marker21354	75	T	C	0.053	6.774E-09	17.212	5.933	—	
	Marker21402	41	C	T	0.170	1.578E-08	12.882	−5.757	−6.350	
	Marker38381	165	T	A	0.070	1.835E-08	13.235	10.858	−11.726	
	Marker26040	148	C	T	0.062	2.722E-08	13.150	−5.992	—	
	Marker22793	161	T	A	0.050	3.006E-08	12.440	10.809	−11.111	
	Marker19793	48	G	A	0.058	3.227E-08	12.331	8.984	−9.881	
	Marker68088	184	G	A	0.040	4.068E-08	17.652	5.446	—	
	Marker22827	88	T	C	0.057	4.723E-08	13.795	4.714	—	
	Marker26994	21	G	A	0.050	4.877E-08	12.070	—	—	
	Marker28210	173	G	A	0.055	5.352E-08	12.861	10.774	—	
	Marker45711	150	G	A	0.080	5.382E-08	15.396	—	12.595	
	Marker32230	180	G	A	0.070	5.820E-08	11.859	10.068	−11.089	
	Marker23996	145	C	T	0.067	7.985E-08	11.670	−10.056	—	
	Marker24465	5	T	G	0.173	8.670E-08	11.658	6.142	—
Apigenin	Marker21022	4	G	C	0.123	1.570E-24	44.750	1.877	−1.885
	Marker21022	172	G	A	0.122	5.820E-17	34.679	1.708	−1.715
	Marker22660	151	C	T	0.058	3.530E-14	25.283	−2.588	−2.423
	Marker20748	85	G	A	0.057	3.570E-14	23.159	2.580	−2.440
	Marker31865	188	T	G	0.052	3.970E-14	28.949	2.543	−2.712
	Marker31467	166	G	A	0.053	4.090E-14	23.007	2.591	−2.474
	Marker31467	190	G	C	0.088	5.180E-14	22.813	2.581	−2.527
	Marker37949	139	G	A	0.045	8.560E-14	30.848	2.578	−2.507
	Marker23202	152	G	C	0.062	1.090E-13	25.641	2.549	−2.687
	Marker24559	31	G	C	0.058	1.330E-13	22.022	1.851	−1.777
	Marker27425	37	C	T	0.053	6.590E-12	18.948	−2.321	−2.395
	Marker21022	171	A	G	0.327	1.100E-11	18.517	−0.795	−0.807
	Marker24949	197	T	G	0.080	2.730E-11	17.750	1.651	−1.737
	Marker36953	169	C	G	0.057	3.450E-11	17.696	−2.416	−2.542
	Marker166717	48	G	A	0.058	3.820E-11	17.572	2.420	−2.507
	Marker27288	176	A	G	0.058	5.300E-11	17.932	−2.426	−2.368
	Marker25068	129	G	T	0.037	9.740E-11	20.651	—	1.493
	Marker40944	148	A	G	0.058	2.950E-10	16.539	−0.999	—
	Marker21022	162	A	G	0.385	9.820E-10	14.988	−0.632	−0.696
	Marker21022	118	C	T	0.388	1.160E-09	14.848	−0.623	−0.695
	Marker21022	197	A	G	0.388	1.690E-09	14.561	−0.614	−0.682
	Marker19793	48	G	A	0.058	7.800E-09	13.417	1.211	−1.322
	Marker38907	186	G	A	0.060	1.270E-08	13.407	0.896	—
	Marker68088	184	G	A	0.040	1.270E-08	19.500	0.719	—
	Marker46448	143	T	G	0.047	2.990E-08	14.165	0.808	—
	Marker22827	88	T	C	0.057	3.210E-08	14.137	0.621	—
	Marker42780	185	C	A	0.055	3.480E-08	14.923	0.805	—
	Marker71813	204	A	T	0.053	3.970E-08	12.915	—	—
	Marker21402	41	C	T	0.170	4.780E-08	12.059	−0.707	−0.787
	Marker26994	21	G	A	0.050	6.200E-08	11.884	—	—
	Marker57496	83	T	G	0.047	6.600E-08	12.041	—	1.595
	Marker31467	164	T	C	0.090	7.900E-08	11.603	1.299	—
	Marker21354	75	T	C	0.053	8.600E-08	15.527	0.699	—
	Marker26040	148	C	T	0.062	9.110E-08	12.166	−0.739	—

*p* < 1E-7.

### Genotype Effects of Most Significantly Associated SNP With Each Traits

To further analyze the genotype effect of key loci, we selected the five SNP loci with the smallest *p* value, representing the five SNP loci with the most significant associations, and assigned them to perform genotype effect analysis (Figures 3, 4). By analyzing the genotype effect of the five associated SNP loci, we found that the homozygous and heterozygous genotypes showed different degrees of significant differences in association with growth-related traits and secondary metabolite contents.

**Figure 5:**
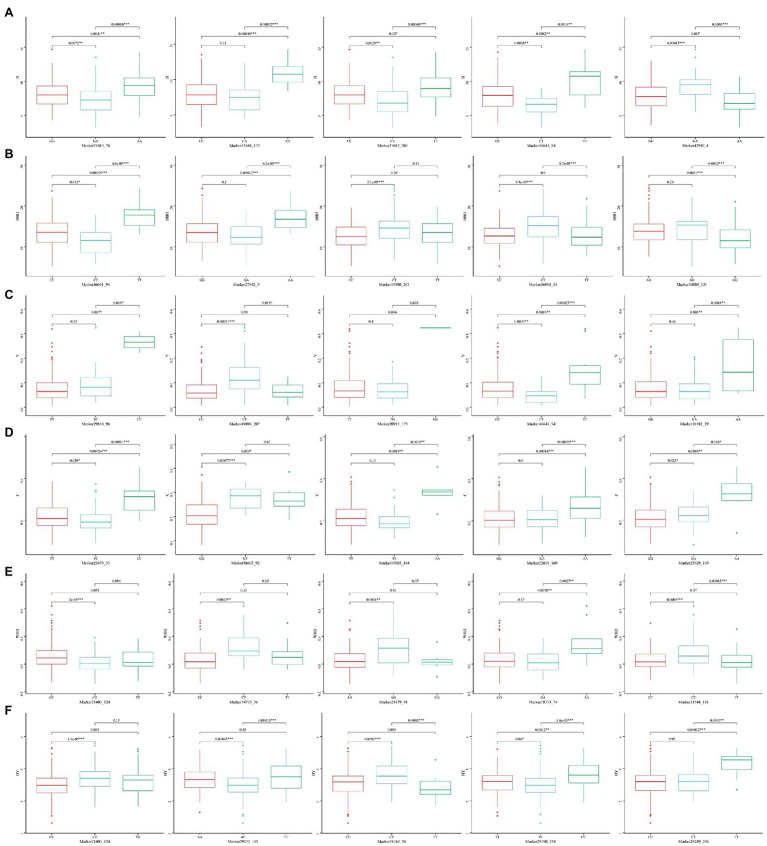
Box plots showing genotype effects of the top five single nucleotide polymorphisms (SNPs) associated with growth-related traits. **(A)** H, **(B)** DBH, **(C)** V, **(D)** P, **(E)** WBD, and **(F)** HY.

**Figure 6:**
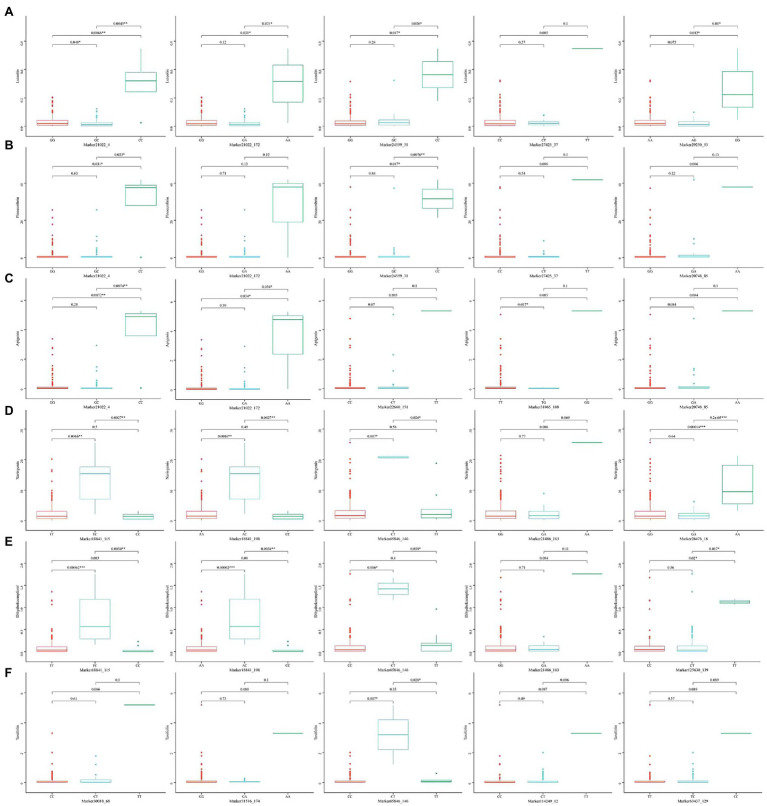
Box plots showing genotype effects of the top five SNPs associated with secondary metabolite contents. **(A)** Luteolin, **(B)** pinocembrin, **(C)** apigenin, **(D)** naringenin, **(E)** DK, and **(F)** DQ.

The five SNP loci that were most significantly associated with each trait were selected and analyzed for genotype effects (Figures 5, 6). Overall, we identified differences among the SNP marker loci significantly associated with each growth-related trait, with only two loci found to be associated with multiple traits, including Marker46641_94 in H, DBH, and V, and Marker21403_124 in WBD and HY. For Marker46641_94, accessions with the homozygous TT genotype were associated with higher H, DBH, and V values, indicating a possible association with the growth of Chinese fir. For Marker21403_124, accessions with the heterozygous CT genotype were associated with a lower WBD and higher HY, indicating a possible association with wood quality. In association study with secondary metabolite contents, there were more single loci correlated with multiple traits, including Marker21022_4 and Marker21022_172 in luteolin, pinocembrin, and apigenin; Marker24559_31 and Marker27425_37 in luteolin and pinocembrin; Marker20748_85 in pinocembrin and apigenin; Marker18841_115, Marker18841_198, and Marker21486_163 in naringenin and DK; and Marker65846_146 in naringenin, DK, and DQ. For Marker21022_4 and Marker21022_172, accessions with homozygous CC and AA, respectively, were associated with higher luteolin, pinocembrin, and apigenin contents, and accessions with homozygous CC, TT, and AA in Marker24559_31, Marker27425_37, and Marker20748_85 showed the same trend. Marker18841_115, Marker18841_198, and Marker65846_146 accessions with heterozygous TC, AC, and CT, respectively, were associated with a higher content of naringenin and DK. In addition, accessions with heterozygous CT in Marker65846_146 were associated with higher DQ content. Moreover, for Marker21486_163, accessions with homozygous AA were associated with a higher content of DQ.

## Discussion

Growth-related traits are important phenotypic traits of forest trees and are closely related to wood quality. Breeders have been working to improve wood quality by enhancing these traits. Secondary metabolites play a crucial role in heartwood, not only affecting the corrosion resistance and insect resistance of most woody plant heartwood, but also producing color substances through chemical reactions that bring coloration to the heartwood, further improving its marketability. Through the difference analysis of the 12 phenotypic traits in 300 Chinese fir clones, we reported that RH grew more slowly but the wood quality was higher when compared with that of WH Chinese fir. Moreover, analyzing the ratios of maximum and minimum measurements of the growth-related traits, we found that the phenotype of RH changes less, the characteristics are more stable, and the growth is more uniform. The determination of the correlation of the six traits with the content of secondary metabolites revealed that the contents of DK, DQ, and naringenin were higher in RH. DK, DQ, and naringenin belong to the middle and upper metabolites in the flavonoid biosynthesis pathway, and a higher content indicates more sufficient substrates for the synthesis of downstream products. In the middle and lower reaches of the pathway, the lower content of luteolin, pinocembrin, and apigenin in RH may be due to glycosylation and other processes, resulting in more stable glycosylation products, indicating that the glycosylation process in RH may also be more active than that in WH. Compared with WH, RH has more upstream substrates and downstream glycosylation products, which ultimately changes the color of heartwood. In a previous study, we found that the differentially expressed genes in phenylalanine pathway (PAL, C4H, and 4CL), upstream of flavonoid biosynthesis pathway (CHS, CHI and F3H) and glycosylation (UGT) were all up-regulated in the transition zone of RH compare to WH (Cao et al., 2022).

By analyzing the 12 phenotypic traits of the 300 core populations screened, it was shown that the traits of this population were little affected by the environment and can be inherited stably (Supplementary Table S1). H and DBH represent the growth rate of woody plants and V represents the yield of wood, which can clarify the growth advantage of the clones. WBD is another important indicator of wood growth-related traits that can affect the final wood quality. Wood density varies widely among different individuals and generally has strong heritability, which is weakly affected by environmental factors (Louzada and Fonseca, 2002). Percentage of heartwood (P), which represents the yield of heartwood, is an important indicator for evaluating the growth of Chinese fir. In the present study, no significant correlations were found with other growth-related traits. In previous studies, it was demonstrated that a large amount of metabolic activity occurred in the transition zone of xylem, with the death of ray parenchyma cells and the disappearance of starch grains, the transition zone would transform into heartwood (Nakaba et al., 2012; Cao et al., 2022). HY refers to the weight increase of the wood after it is completely soaked in water, which is affected by WBD. In this study, a significant negative correlation with WBD was detected, which was consistent with the results of other studies (Duan et al., 2016). We found that most growth-related traits had significant positive correlations between pairs, with the exception of WBD, which had a significant negative correlation compared with H, DBH, V, and HY, and was consistent with previous literature (Duan et al., 2016). In addition, there was no significant correlation between P and other phenotypes in this study, and similar trends have rarely been reported in other species.

GWAS are observational studies of a set of genome-wide genetic variants in the populations and their association with specific traits. Recent years, GWAS have been shown to be very powerful in analyzing the genetic basis of complex phenotypic variation, primarily applied to the study of physiological and agricultural traits in plants (Huang et al., 2010; Tian et al., 2011; Horton et al., 2012; Chen et al., 2016; Yano et al., 2016; Xiao et al., 2017). In a MLM, the population structure and kinship are comprehensively analyzed, making it the most common used GWAS method. Besides, it also has demonstrated high efficiency in correcting for inflation caused by many small genetic effects, and for controlling for bias in population stratification (Yu et al., 2006; Zhao et al., 2007). In our investigation, to reduce the occurrence of false-positive results in association analysis, the MLM model in TASSEL5.0, was used. The qq plot showed that MLM was suitable for the association analysis of Chinese fir growth-related traits and secondary metabolite contents; however, if the same threshold was used (*p* < 1E-4), the number of association sites for secondary metabolite contents was significantly higher than that for growth-related traits. Therefore, we set the significant association threshold for secondary metabolites to *p* < 1E-7 and obtained SNP sites with more significant associations. Using MLM, we obtained 62 SNP marker loci related to growth-related traits and 163 SNP marker loci related to secondary metabolite contents. Since there may be corresponding genes near the obtained association loci (Morris et al., 2013), the genes containing these SNPs loci may be involved in the growth of fir or the synthesis of flavonoid secondary metabolites. Palle et al. (2013) used the SNPs markers to analyze the association between the expression levels of genes potentially related to loblolly pine wood formation, thus contributing to a deeper understanding of phenotypic variation in Chinese fir. In addition, with the rapid development of sequencing technology, an increasing number of tree species, including Chinese fir, will undergo whole-genome sequencing, which will help us to further explain the associated loci obtained in this study.

The GWAS performed using MLM had additive or dominant effects or both. This indicated that the phenotypic of heterozygous Chinese fir may be better than those of homozygous samples, which was also proved by the SNPs-phenotype correlation analysis. For example, Marker42910_4 was related to H, Marker36988_54 was related to DBH, Marker49894_207 was related to V, Marker31544_181 was related to WBD, and Marker21169_24 was related to HY. It was found that for heterozygous individuals at these loci in the related population, the numerical mean of heterozygous was significantly higher than that of homozygous individuals, and these loci with dominant or additive effects may be the important genetic basis for heterosis in Chinese fir. We also observed that the same marker was associated with multiple traits, primarily secondary metabolites. For example, Marker21022_4 and Marker21022_172 co-existed in association with luteolin, pinocembrin, and apigenin, whereas Marker18841_115, Marker18841_198, Marker65846_146, and Marker21486_163 were also significantly associated with naringenin and DK, and most of the significant association loci in DQ were special. Therefore, we also divided the detected flavonoid secondary metabolites into three categories according to the level of significant association, which indicated that the flavonoid metabolites may have different regulatory genes owing to their different chemical structures. In recent years, there have been many reports of GWAS associated with metabolism. Xiao et al. (2021) identified eight quantitative trait loci significantly associated with the metabolic pathway of Salicylic acid (SA) biosynthesis in *Populus*. During GWAS of tea, it was found that 307 SNP marker were proved to be related to some flavor-related metabolites such as theanine, caffeine and catechins, and some of the markers were even pleiotropic (Fang et al., 2021). In the present study, some single markers are associated with multiple traits and illustrate the close correlation between secondary metabolite traits, indicating pleiotropic effects, and reflecting the complexity of physiological and biochemical metabolic processes. This phenomenon has been reported in several studies. For example, Tian et al. found that the SNP10 locus located in the *PtGA20Ox* gene was significantly associated with one wood growth trait and three texture traits, simultaneously (Tian et al., 2012). Yang et al. (2015) also found that the SNP11 locus located in the *Pto-Wuschela* gene was significantly associated with tree height and volume per plant. The sites associated with multiple traits may be more effective in molecular marker-assisted selection and may also be helpful for mining important genomic regions.

## Conclusion

This study found that there were differences in growth between white- and red-heart Chinese fir. Red-heart Chinese fir grows more slowly but the wood quality is higher. The 12 traits of this population were little affected by the environment and can be inherited stably. Furthermore, several single SNP loci which significantly associated with secondary metabolite contents has been found, indicating pleiotropic effects. Chinese fir is a perennial conifer with a huge genome. As of this manuscript writing, its genome has not been published. At present, it is impossible to locate SNP loci in chromosomes and genes by simplified GWAS. In the future research, the loci can be accurately located with reference to the genome to promote the process of molecular marker-assisted breeding of Chinese fir.

## Data Availability Statement

The datasets presented in this study can be found in online repositories. The names of the repository/repositories and accession number(s) can be found at: We have uploaded the data on GSA data base at https://ngdc.cncb.ac.cn/, and the GSA accession number is CRA007096.

## Author Contributions

YL and HZ conceived and designed the experiments. SC wrote the article. SC, HD, and BW performed the experiments. SC, YS, and RH analyzed the data. JL and WD participated in and helped to complete the experiments. All authors contributed to the article and approved the submitted version.

## Funding

This research was supported by the Key-Area Research and Development Program of Guangdong Province (no. 2020B020215001), the Science and Technology Research Project of Beijing Forestry University (2018WS01), and the National Natural Science Foundation of China (no. 31972956).

## Conflict of Interest

The authors declare that the research was conducted in the absence of any commercial or financial relationships that could be construed as a potential conflict of interest.

## Publisher’s Note

All claims expressed in this article are solely those of the authors and do not necessarily represent those of their affiliated organizations, or those of the publisher, the editors and the reviewers. Any product that may be evaluated in this article, or claim that may be made by its manufacturer, is not guaranteed or endorsed by the publisher.

## Supplementary Material

The Supplementary Material for this article can be found online at: https://www.frontiersin.org/articles/10.3389/fpls.2022.922007/full#supplementary-material

Supplementary Table S1 The variance components and the generalized heritability of various characters in associated group.

Supplementary Table S2 The SNP information.
