# Supplementary material for: Genome-Wide Association Study With Growth-Related Traits and Secondary Metabolite Contents in Red- and White-Heart Chinese Fir
Source: Front Plant Sci. 2022 Jun 30;13:922007. doi: 10.3389/fpls.2022.922007 (PMC9280351; doi:10.3389/fpls.2022.922007)
Supplement: Supplementary Table S1 — The variance components and the generalized heritability of various characters in associated group. [file Data_Sheet_1.docx]

Table S1 The variance components and the generalized heritability of various characters in associated group.

|  | H | DBH | V | C | WBD | HY | DK | DQ | Luteolin | Pinocembrin | Apigenin | Naringenin |
| --- | --- | --- | --- | --- | --- | --- | --- | --- | --- | --- | --- | --- |
| V_G_ | 3.46 | 9.16 | 2.35E-3 | 1.55E-3 | 1.25E-3 | 0.11 | 0.16 | 0.50 | 0.02 | 75.16 | 1.23 | 44.26 |
| V_E_ | 0.03 | 0.00 | 0.00 | 1.31E-5 | 0.00 | 0.00 | 0.00 | 0.00 | 0.00 | 0.89 | 0.01 | 0.03 |
| Residual | 2.23 | 7.18 | 1.70E-3 | 8.67E-3 | 1.73E-3 | 0.15 | 0.05 | 0.17 | 0.01 | 25.32 | 0.41 | 14.76 |
| H_B_^2^/% | 60.49 | 56.06 | 58.02 | 15.15 | 41.95 | 42.31 | 76.19 | 75.01 | 74.07 | 74.15 | 74.35 | 74.96 |

H: Height, DBH: diameter at breast height, V: stem volume, P: percentage of heartwood, WBD: wood basic density, HY: hygroscopicity, DK: Dihydrokaempferol, DQ: Dihydroquercetin (Taxifolin). V_G_: genetic variance, V_E_: environment variance, H_B_^2^: generalized heritability

Table S2 The SNP information

| Client _ID | BMK _ID | Total SNP | SNP number | Heter -ratio | Integrity -ratio | Client _ID | BMK _ID | Total SNP | SNP number | Heter -ratio | Integrity -ratio |
| --- | --- | --- | --- | --- | --- | --- | --- | --- | --- | --- | --- |
| 1 | aa | 955503 | 324340 | 7.25% | 33.94% | 246 | fu | 955503 | 323058 | 6.24% | 33.81% |
| 6 | ab | 955503 | 375019 | 7.47% | 39.24% | 250 | fv | 955503 | 359877 | 6.74% | 37.66% |
| 11 | ac | 955503 | 406762 | 8.68% | 42.57% | 252 | fw | 955503 | 379896 | 6.77% | 39.75% |
| 3 | ad | 955503 | 290133 | 7.86% | 30.36% | 253 | fx | 955503 | 369425 | 7.14% | 38.66% |
| 7 | ae | 955503 | 417193 | 8.46% | 43.66% | 255 | fy | 955503 | 378671 | 7.21% | 39.63% |
| 8 | af | 955503 | 333632 | 6.84% | 34.91% | 256 | fz | 955503 | 441685 | 8.45% | 46.22% |
| 9 | ag | 955503 | 414410 | 8.66% | 43.37% | 258 | ga | 955503 | 377383 | 7.23% | 39.49% |
| 97 | ah | 955503 | 363387 | 7.14% | 38.03% | 263 | gb | 955503 | 373891 | 7.67% | 39.13% |
| 12 | ai | 955503 | 384846 | 7.80% | 40.27% | 266 | gc | 955503 | 333760 | 6.25% | 34.93% |
| 13 | aj | 955503 | 338738 | 6.44% | 35.45% | 267 | gd | 955503 | 358221 | 6.74% | 37.49% |
| 14 | ak | 955503 | 395418 | 8.04% | 41.38% | 269 | ge | 955503 | 349963 | 7.46% | 36.62% |
| 167 | al | 955503 | 420599 | 8.39% | 44.01% | 272 | gf | 955503 | 359204 | 7.03% | 37.59% |
| 15 | am | 955503 | 370951 | 7.20% | 38.82% | 273 | gg | 955503 | 382549 | 7.80% | 40.03% |
| 16 | an | 955503 | 403610 | 8.02% | 42.24% | 276 | gh | 955503 | 376977 | 7.16% | 39.45% |
| 245 | ao | 955503 | 418580 | 8.01% | 43.80% | 278 | gi | 955503 | 374209 | 7.22% | 39.16% |
| 18 | ap | 955503 | 429032 | 8.66% | 44.90% | 282 | gj | 955503 | 379176 | 6.89% | 39.68% |
| 259 | aq | 955503 | 237642 | 7.23% | 24.87% | 284 | gk | 955503 | 385168 | 7.76% | 40.31% |
| 277 | ar | 955503 | 357403 | 6.83% | 37.40% | 285 | gl | 955503 | 393883 | 7.93% | 41.22% |
| 19 | as | 955503 | 353195 | 7.12% | 36.96% | 289 | gm | 955503 | 374653 | 7.12% | 39.21% |
| 20 | at | 955503 | 375428 | 7.50% | 39.29% | 290 | gn | 955503 | 379435 | 7.54% | 39.71% |
| 23 | au | 955503 | 404385 | 7.99% | 42.32% | 295 | go | 955503 | 367252 | 8.50% | 38.43% |
| 21 | av | 955503 | 381407 | 7.66% | 39.91% | 296 | gp | 955503 | 371695 | 7.77% | 38.90% |
| 62 | aw | 955503 | 394114 | 7.73% | 41.24% | 297 | gq | 955503 | 352755 | 6.87% | 36.91% |
| 337 | ax | 955503 | 303733 | 6.35% | 31.78% | 301 | gr | 955503 | 386884 | 7.27% | 40.49% |
| 68 | ay | 955503 | 340646 | 6.79% | 35.65% | 305 | gs | 955503 | 335631 | 5.92% | 35.12% |
| 95 | az | 955503 | 379492 | 7.66% | 39.71% | 306 | gt | 955503 | 370156 | 6.26% | 38.73% |
| 26 | ba | 955503 | 261539 | 6.68% | 27.37% | 308 | gu | 955503 | 371280 | 7.48% | 38.85% |
| 27 | bb | 955503 | 391637 | 7.38% | 40.98% | 312 | gv | 955503 | 337021 | 6.14% | 35.27% |
| 101 | bc | 955503 | 434779 | 8% | 45.50% | 313 | gw | 955503 | 350917 | 7.39% | 36.72% |
| 348 | bd | 955503 | 285827 | 7.46% | 29.91% | 315 | gx | 955503 | 362448 | 7.47% | 37.93% |
| 29 | be | 955503 | 342571 | 7.49% | 35.85% | 319 | gy | 955503 | 360537 | 8.97% | 37.73% |
| 104 | bf | 955503 | 375653 | 7.33% | 39.31% | 321 | gz | 955503 | 358977 | 7.09% | 37.56% |
| 369 | bg | 955503 | 412826 | 8.22% | 43.20% | 325 | ha | 955503 | 353019 | 6.83% | 36.94% |
| 32 | bh | 955503 | 257206 | 6.60% | 26.91% | 327 | hb | 955503 | 340349 | 6.57% | 35.61% |
| 218 | bi | 955503 | 414735 | 7.63% | 43.40% | 328 | hc | 955503 | 342783 | 6.37% | 35.87% |
| 370 | bj | 955503 | 389086 | 7.79% | 40.72% | 331 | hd | 955503 | 328447 | 5.86% | 34.37% |
| 34 | bk | 955503 | 395177 | 8.02% | 41.35% | 332 | he | 955503 | 272142 | 5.61% | 28.48% |
| 286 | bl | 955503 | 406849 | 7.55% | 42.57% | 333 | hf | 955503 | 363526 | 7.79% | 38.04% |
| 398 | bm | 955503 | 376383 | 7.44% | 39.39% | 334 | hg | 955503 | 346205 | 6.75% | 36.23% |
| 39 | bn | 955503 | 369103 | 6.94% | 38.62% | 335 | hh | 955503 | 367446 | 9.74% | 38.45% |
| 292 | bo | 955503 | 377723 | 7.60% | 39.53% | 336 | hi | 955503 | 370945 | 7.29% | 38.82% |
| 406 | bp | 955503 | 234148 | 6.82% | 24.50% | 338 | hj | 955503 | 321688 | 6.95% | 33.66% |
| 41 | bq | 955503 | 402490 | 7.90% | 42.12% | 341 | hk | 955503 | 322320 | 5.88% | 33.73% |
| 347 | br | 955503 | 390970 | 7.93% | 40.91% | 342 | hl | 955503 | 330538 | 5.97% | 34.59% |
| 423 | bs | 955503 | 258685 | 6.56% | 27.07% | 343 | hm | 955503 | 392354 | 8.06% | 41.06% |
| 42 | bt | 955503 | 370644 | 7.20% | 38.79% | 345 | hn | 955503 | 319830 | 6.48% | 33.47% |
| 421 | bu | 955503 | 405418 | 7.76% | 42.42% | 346 | ho | 955503 | 370406 | 7.21% | 38.76% |
| 427 | bv | 955503 | 379612 | 8.20% | 39.72% | 353 | hp | 955503 | 405169 | 8.11% | 42.40% |
| 43 | bw | 955503 | 423442 | 8.01% | 44.31% | 355 | hq | 955503 | 340771 | 6.90% | 35.66% |
| 442 | bx | 955503 | 453272 | 8.70% | 47.43% | 360 | hr | 955503 | 372556 | 7.69% | 38.99% |
| 503 | by | 955503 | 375576 | 7.14% | 39.30% | 364 | hs | 955503 | 377849 | 7.49% | 39.54% |
| 45 | bz | 955503 | 278199 | 6.87% | 29.11% | 373 | ht | 955503 | 354493 | 6.30% | 37.10% |
| 46 | ca | 955503 | 395022 | 7.58% | 41.34% | 383 | hu | 955503 | 375657 | 7.76% | 39.31% |
| 461 | cb | 955503 | 423809 | 8.49% | 44.35% | 385 | hv | 955503 | 380273 | 7.36% | 39.79% |
| 524 | cc | 955503 | 391394 | 7.12% | 40.96% | 388 | hw | 955503 | 377764 | 7.18% | 39.53% |
| 564 | cd | 955503 | 389419 | 7.25% | 40.75% | 391 | hx | 955503 | 271713 | 6.58% | 28.43% |
| 49 | ce | 955503 | 401061 | 7.88% | 41.97% | 394 | hy | 955503 | 400289 | 8.90% | 41.89% |
| 473 | cf | 955503 | 396824 | 7.16% | 41.53% | 399 | hz | 955503 | 370072 | 7.24% | 38.73% |
| 592 | cg | 955503 | 405989 | 7.76% | 42.48% | 400 | ia | 955503 | 372100 | 7.42% | 38.94% |
| 52 | ch | 955503 | 404185 | 7.46% | 42.30% | 403 | ib | 955503 | 359847 | 7.27% | 37.66% |
| 490 | ci | 955503 | 390183 | 7.48% | 40.83% | 404 | ic | 955503 | 380871 | 7.42% | 39.86% |
| 57 | cj | 955503 | 392368 | 7.70% | 41.06% | 405 | id | 955503 | 384686 | 7.09% | 40.26% |
| 618 | ck | 955503 | 402625 | 7.54% | 42.13% | 407 | ie | 955503 | 416321 | 7.65% | 43.57% |
| 493 | cl | 955503 | 418232 | 7.49% | 43.77% | 408 | if | 955503 | 373112 | 7.15% | 39.04% |
| 495 | cm | 955503 | 397757 | 7.85% | 41.62% | 409 | ig | 955503 | 380194 | 7.35% | 39.78% |
| 59 | cn | 955503 | 400081 | 7.61% | 41.87% | 410 | ih | 955503 | 397246 | 7.37% | 41.57% |
| 654 | co | 955503 | 390162 | 7.30% | 40.83% | 412 | ii | 955503 | 364722 | 6.72% | 38.17% |
| 67 | cp | 955503 | 380933 | 7.17% | 39.86% | 414 | ij | 955503 | 374345 | 6.64% | 39.17% |
| 506 | cq | 955503 | 325690 | 6.80% | 34.08% | 419 | ik | 955503 | 319823 | 6.96% | 33.47% |
| 507 | cr | 955503 | 385773 | 7.01% | 40.37% | 420 | il | 955503 | 392936 | 8.42% | 41.12% |
| 70 | cs | 955503 | 407456 | 8.16% | 42.64% | 425 | im | 955503 | 325820 | 6.46% | 34.09% |
| 525 | ct | 955503 | 436972 | 10.28% | 45.73% | 426 | in | 955503 | 370391 | 8.12% | 38.76% |
| 71 | cu | 955503 | 395113 | 7.91% | 41.35% | 428 | io | 955503 | 381480 | 8.17% | 39.92% |
| 538 | cv | 955503 | 409997 | 7.42% | 42.90% | 430 | ip | 955503 | 329650 | 6.81% | 34.50% |
| 75 | cw | 955503 | 317069 | 5.61% | 33.18% | 432 | iq | 955503 | 345196 | 7.07% | 36.12% |
| 77 | cx | 955503 | 429140 | 8.01% | 44.91% | 434 | ir | 955503 | 360110 | 7.40% | 37.68% |
| 561 | cy | 955503 | 294129 | 7.23% | 30.78% | 435 | is | 955503 | 390001 | 7.96% | 40.81% |
| 80 | cz | 955503 | 369231 | 7.25% | 38.64% | 436 | it | 955503 | 401130 | 7.92% | 41.98% |
| 566 | da | 955503 | 396599 | 7.67% | 41.50% | 437 | iu | 955503 | 409393 | 8.36% | 42.84% |
| 81 | db | 955503 | 411245 | 8.43% | 43.03% | 439 | iv | 955503 | 420796 | 8.62% | 44.03% |
| 82 | dc | 955503 | 399591 | 7.81% | 41.81% | 440 | iw | 955503 | 250365 | 6.92% | 26.20% |
| 576 | dd | 955503 | 229274 | 6.25% | 23.99% | 445 | ix | 955503 | 375958 | 7.41% | 39.34% |
| 83 | de | 955503 | 359596 | 7.19% | 37.63% | 446 | iy | 955503 | 375471 | 7.19% | 39.29% |
| 582 | df | 955503 | 378904 | 7.45% | 39.65% | 449 | iz | 955503 | 373581 | 7.19% | 39.09% |
| 85 | dg | 955503 | 250213 | 5.91% | 26.18% | 455 | ja | 955503 | 370241 | 6.95% | 38.74% |
| 593 | dh | 955503 | 364371 | 6.57% | 38.13% | 456 | jb | 955503 | 383935 | 7.82% | 40.18% |
| 86 | di | 955503 | 354274 | 6.39% | 37.07% | 465 | jc | 955503 | 331929 | 6.55% | 34.73% |
| 598 | dj | 955503 | 390865 | 7.85% | 40.90% | 474 | jd | 955503 | 367942 | 7.09% | 38.50% |
| 90 | dk | 955503 | 378105 | 7.23% | 39.57% | 475 | je | 955503 | 377963 | 6.92% | 39.55% |
| 92 | dl | 955503 | 388187 | 7.43% | 40.62% | 477 | jf | 955503 | 387039 | 8.04% | 40.50% |
| 602 | dm | 955503 | 340783 | 5.62% | 35.66% | 478 | jg | 955503 | 368919 | 7.44% | 38.60% |
| 607 | dn | 955503 | 389109 | 6.68% | 40.72% | 488 | jh | 955503 | 374311 | 7.23% | 39.17% |
| 608 | do | 955503 | 377226 | 7.01% | 39.47% | 492 | ji | 955503 | 345572 | 6.91% | 36.16% |
| 610 | dp | 955503 | 375306 | 7.15% | 39.27% | 494 | jj | 955503 | 346012 | 6.57% | 36.21% |
| 612 | dq | 955503 | 369920 | 7.18% | 38.71% | 496 | jk | 955503 | 354369 | 6.59% | 37.08% |
| 107 | dr | 955503 | 358985 | 6.63% | 37.57% | 512 | jl | 955503 | 378001 | 7.91% | 39.56% |
| 110 | ds | 955503 | 361397 | 6.75% | 37.82% | 514 | jm | 955503 | 398658 | 9.86% | 41.72% |
| 645 | dt | 955503 | 352614 | 7% | 36.90% | 520 | jn | 955503 | 379834 | 7.65% | 39.75% |
| 113 | du | 955503 | 386959 | 7.76% | 40.49% | 521 | jo | 955503 | 374753 | 7.51% | 39.22% |
| 655 | dv | 955503 | 363152 | 6.64% | 38% | 529 | jp | 955503 | 364212 | 6.71% | 38.11% |
| 125 | dw | 955503 | 410814 | 7.47% | 42.99% | 535 | jq | 955503 | 363293 | 7% | 38.02% |
| 673 | dx | 955503 | 261948 | 6.35% | 27.41% | 536 | jr | 955503 | 304135 | 6.67% | 31.82% |
| 131 | dy | 955503 | 370951 | 7.33% | 38.82% | 544 | js | 955503 | 378377 | 7.19% | 39.59% |
| 132 | dz | 955503 | 359173 | 6.67% | 37.58% | 553 | jt | 955503 | 374223 | 7.43% | 39.16% |
| 693 | ea | 955503 | 347752 | 6.66% | 36.39% | 563 | ju | 955503 | 343068 | 7.03% | 35.90% |
| 135 | eb | 955503 | 383441 | 7.35% | 40.12% | 565 | jv | 955503 | 351677 | 8.27% | 36.80% |
| 714 | ec | 955503 | 380901 | 7.29% | 39.86% | 567 | jw | 955503 | 360635 | 6.80% | 37.74% |
| 137 | ed | 955503 | 395557 | 6.85% | 41.39% | 569 | jx | 955503 | 388575 | 7.65% | 40.66% |
| 717 | ee | 955503 | 278295 | 6.63% | 29.12% | 571 | jy | 955503 | 363171 | 6.49% | 38% |
| 138 | ef | 955503 | 374066 | 7.27% | 39.14% | 572 | jz | 955503 | 380057 | 7.83% | 39.77% |
| 725 | eg | 955503 | 379663 | 7.61% | 39.73% | 573 | ka | 955503 | 367347 | 7.63% | 38.44% |
| 140 | eh | 955503 | 369826 | 6.86% | 38.70% | 574 | kb | 955503 | 399457 | 7.19% | 41.80% |
| 727 | ei | 955503 | 418501 | 8.03% | 43.79% | 575 | kc | 955503 | 406054 | 7.80% | 42.49% |
| 145 | ej | 955503 | 364077 | 6.67% | 38.10% | 580 | kd | 955503 | 367100 | 7.09% | 38.41% |
| 146 | ek | 955503 | 384617 | 7.54% | 40.25% | 585 | ke | 955503 | 372117 | 7.11% | 38.94% |
| 148 | el | 955503 | 332724 | 6.33% | 34.82% | 591 | kf | 955503 | 416557 | 7.55% | 43.59% |
| 150 | em | 955503 | 412943 | 7.53% | 43.21% | 597 | kg | 955503 | 434350 | 7.97% | 45.45% |
| 154 | en | 955503 | 376183 | 7.48% | 39.37% | 601 | kh | 955503 | 360667 | 7.84% | 37.74% |
| 155 | eo | 955503 | 388159 | 7.53% | 40.62% | 603 | ki | 955503 | 379407 | 7.44% | 39.70% |
| 156 | ep | 955503 | 385474 | 7.43% | 40.34% | 611 | kj | 955503 | 389825 | 8.09% | 40.79% |
| 160 | eq | 955503 | 396833 | 7.86% | 41.53% | 613 | kk | 955503 | 370306 | 7.25% | 38.75% |
| 169 | er | 955503 | 372158 | 7.11% | 38.94% | 616 | kl | 955503 | 375663 | 7.78% | 39.31% |
| 172 | es | 955503 | 359721 | 6.91% | 37.64% | 620 | km | 955503 | 371429 | 7.41% | 38.87% |
| 174 | et | 955503 | 391977 | 7.76% | 41.02% | 626 | kn | 955503 | 380436 | 8.31% | 39.81% |
| 177 | eu | 955503 | 237358 | 6.44% | 24.84% | 628 | ko | 955503 | 371702 | 7.27% | 38.90% |
| 182 | ev | 955503 | 386967 | 7.26% | 40.49% | 631 | kp | 955503 | 368585 | 8.06% | 38.57% |
| 189 | ew | 955503 | 378026 | 7.41% | 39.56% | 633 | kq | 955503 | 403878 | 8.44% | 42.26% |
| 190 | ex | 955503 | 377373 | 7.64% | 39.49% | 637 | kr | 955503 | 368035 | 7.50% | 38.51% |
| 195 | ey | 955503 | 366817 | 6.90% | 38.38% | 640 | ks | 955503 | 294799 | 5.98% | 30.85% |
| 196 | ez | 955503 | 359760 | 6.79% | 37.65% | 643 | kt | 955503 | 375215 | 7.23% | 39.26% |
| 197 | fa | 955503 | 367883 | 6.51% | 38.50% | 644 | ku | 955503 | 371417 | 7.66% | 38.87% |
| 200 | fb | 955503 | 385895 | 7.40% | 40.38% | 651 | kv | 955503 | 401927 | 7.26% | 42.06% |
| 202 | fc | 955503 | 386834 | 7.29% | 40.48% | 658 | kw | 955503 | 358791 | 7.33% | 37.54% |
| 206 | fd | 955503 | 383822 | 7.40% | 40.16% | 660 | kx | 955503 | 380030 | 8.15% | 39.77% |
| 207 | fe | 955503 | 361156 | 6.66% | 37.79% | 665 | ky | 955503 | 372671 | 7.73% | 39% |
| 208 | ff | 955503 | 367414 | 6.87% | 38.45% | 669 | kz | 955503 | 320304 | 6.58% | 33.52% |
| 211 | fg | 955503 | 369735 | 6.97% | 38.69% | 675 | la | 955503 | 389653 | 7.51% | 40.77% |
| 214 | fh | 955503 | 359067 | 6.60% | 37.57% | 677 | lb | 955503 | 395384 | 7.85% | 41.37% |
| 215 | fi | 955503 | 374065 | 7.45% | 39.14% | 679 | lc | 955503 | 397183 | 7.73% | 41.56% |
| 216 | fj | 955503 | 377105 | 6.34% | 39.46% | 680 | ld | 955503 | 301796 | 6.56% | 31.58% |
| 222 | fk | 955503 | 328541 | 6.31% | 34.38% | 682 | le | 955503 | 374983 | 7.31% | 39.24% |
| 227 | fl | 955503 | 354152 | 6.51% | 37.06% | 703 | lf | 955503 | 377229 | 7.46% | 39.47% |
| 228 | fm | 955503 | 354014 | 6.75% | 37.05% | 706 | lg | 955503 | 364093 | 7.12% | 38.10% |
| 233 | fn | 955503 | 319806 | 5.98% | 33.46% | 708 | lh | 955503 | 378950 | 7.32% | 39.65% |
| 236 | fo | 955503 | 347825 | 6.67% | 36.40% | 721 | li | 955503 | 411726 | 8.23% | 43.08% |
| 237 | fp | 955503 | 348104 | 6.60% | 36.43% | 723 | lj | 955503 | 359462 | 6.79% | 37.62% |
| 238 | fq | 955503 | 374758 | 7.35% | 39.22% | 728 | lk | 955503 | 383378 | 7.58% | 40.12% |
| 240 | fr | 955503 | 368972 | 6.84% | 38.61% | 730 | ll | 955503 | 373318 | 7.82% | 39.07% |
| 242 | fs | 955503 | 360838 | 6.90% | 37.76% | 731 | lm | 955503 | 380338 | 7.40% | 39.80% |
| 244 | ft | 955503 | 381524 | 8.32% | 39.92% | 734 | ln | 955503 | 371154 | 7.41% | 38.84% |

Total SNP: the total number of detected SNPs, SNP number: the number of detected SNPs in corresponding samples, Heter ratio: ratio of SNP heterozygotes, Integrity ratio: integrity of detected SNPs in samples.
